# Word Boundaries Affect Visual Attention in Chinese Reading

**DOI:** 10.1371/journal.pone.0048905

**Published:** 2012-11-09

**Authors:** Xingshan Li, Guojie Ma

**Affiliations:** Key Laboratory of Behavioral Science, Institute of Psychology, Chinese Academy of Sciences, Beijing, China; University of Sydney, Australia

## Abstract

In two experiments, we explored attention deployment during the reading of Chinese words using a probe detection task. In both experiments, Chinese readers saw four simplified Chinese characters briefly, and then a probe was presented at one of the character positions. The four characters constituted either one word or two words of two characters each. Reaction time was shorter when the probe was at the character 2 position than the character 3 position in the two-word condition, but not in the one-word condition. In Experiment 2, there were more trials and the materials were more carefully controlled, and the results replicated that of Experiment 1. These results suggest that word boundary information affects attentional deployment in Chinese reading.

## Introduction

To correctly understand text, readers have to encode the order of the information correctly. Covert attention arguably plays an important role in this. Most western language reading models assume that words are the primary unit of processing in reading, and assume that attentional deployment is constrained by word boundaries [1], [2]. In English, word boundaries can be perceived easily with the help of low-level visual features (i.e., spaces between words). Hence, words are likely to be a primary unit of attentional deployment. However, not all writing systems have spaces between words. For example, Chinese sentences consist of characters that vary in the complexity of their shapes, but each character fits within the same sized rectangular region. A word consists of one, two, three, or more characters, but because there are no spaces between words to mark word boundaries, Chinese readers cannot segment words with low-level visual features. To successfully segment words, they have to rely on high level knowledge such as which sequences of characters are words. Given this special property of Chinese reading, it is not clear whether attention is constrained by word boundary information during Chinese reading.

Some studies suggest that Chinese words might be perceived as a unit (like an object) and thus constrain attentional deployment, just as objects whose boundaries are defined by bottom-up features do. For example, Li and Logan [3] showed Chinese readers four Chinese characters in two rows and two columns. On half of the trials, two characters in a row constituted a word; on the other half, two characters in a column constituted a word. One of the characters changed color from black to green as an attentional cue after the four characters were shown for 1.5 second, and then after 100 ms another character changed color to red as a target. The participants were asked to press a key when the red target appeared and do nothing when the target did not appear. In the cue-invalid trials (i.e., when the cued object was not the target object), reaction times (RTs) were longer when the cue and the target belonged to adjacent characters from different words compared with when they belonged to adjacent characters that formed a word. These results are similar to those found in object-based attention studies [4], which usually use bottom-up defined objects such as rectangles to define the region to be covertly attended to. This suggested that Chinese words could influence attention deployment in the same way as bottom-up defined objects. Consistent with this study, another study showed that a Ternus display (a group of horizontally aligned elements oscillating in apparent motion) consisting of two Chinese characters was more likely to be reported as group motion when the displayed characters constituted a word than when they did not constitute a word [5], suggesting that word knowledge can affect the grouping of visual elements.

Another study showed that word boundary information can affect character perception during Chinese reading [6]. Subjects were shown four characters briefly, and were asked to report as many characters as possible. These four characters constituted a single 4-character word or two 2-character words. The subjects reported all four characters quite accurately in the one-word condition, but could usually only report the two characters belonging to the first word in the two-word condition. Results of Experiment 4 further explored the possible influence of memory load. In that experiment, participants did not report the characters. Instead, they detected whether a specific character was present or not. Hence, memory load was identical for the one-word condition and two-word condition The character detection accuracy was high when the target was at any of the character positions in the one-word condition. However, accuracy was lower when the target was at a position belonging to the second word than belonging to the first word. These results suggested that the word boundary effect was not likely to be caused by memory load difference between the two conditions. In summary, Li et al. [6] clearly showed that word boundary affect character perception. However, it is not clear whether visual attention deployment was affected by word boundary.

The present study was designed to explore whether word boundary information affects attentional deployment in Chinese reading. We showed participants four Chinese characters very briefly, as Li et al. [6] did. The four characters formed a single 4-character word (one-word condition) or two 2-character words (two-word condition). Immediately following the offset of the characters, there was a probe at one of the character positions in some trials. Subjects were asked to press one of two keys on the keyboard to indicate whether there was a probe or not. Unlike Li et al. [6], participants were not required to report the characters, so that the probe detection task was not interfered by another task. The relative response time (RT) of the probe detection task at different positions is usually thought to reflect low-level attentional deployment [7]. A shorter RT at a position suggests that more attention is being deployed at that position. In the current study, there was a word boundary between the second and third character position in the two-word condition, but not in the one-word condition. If word boundary information affects attentional deployment, and if attentional deployment is largely on the first two characters in the two-word condition, then RT should increase greatly at word boundary position (between characters 2 and 3) in the two-word condition, but not in the one-word condition. Considering the influence of visual eccentricity and other factors, RTs might vary as a function of the distance between the probe and the fixation point. However, these factors should affect RTs in a similar way for the two conditions. Hence, if there is RT difference between probes at the second character position and the third character position varies across the two conditions, we can conclude that word boundary information is affecting attentional deployment.

## Experiment 1

### Methods

#### Participants

Forty undergraduate students (24 women and 16 men) from the China Agricultural University with normal or corrected-to-normal vision were paid to participate in the experiment. Their ages ranged from 18 to 26 years, with an average of 22 years, and a standard deviation of 2 years. This study has been approved by the institutional review board of the Institute of Psychology, Chinese Academy of Sciences. Written consent forms have been obtained.

#### Apparatus

Stimulus presentation and response registration were controlled by a personal computer. Stimuli were presented on a 19-inch LCD monitor (Think Vision L197WA) with a resolution of 1440×900 pixels and a refresh rate of 60 Hz. Participants viewed the stimuli about 70 cm from the monitor.

#### Materials

**Figure 1:**
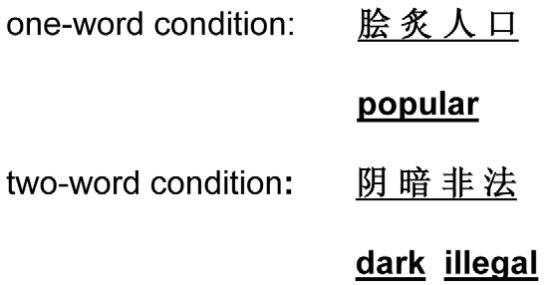
Example stimuli. Note that the lines under the characters were not shown in the experimental displays.

Subjects saw four simplified Chinese characters in a single line on each trial (see Figure 1). In half of the trials, these four characters constituted a 4-character word (the one-word condition). In the other half of the trials, the first two characters constituted a word and the last two constituted another word (two-word condition). Each character fit within a 1°×1° square. There were 10 practice trials, followed by 96 experimental trials. The order of the experimental trials was randomized for each subject. Each of the words was used only once. Character properties are shown in Table 1. The characters were shown in black (RGB: [0, 0, 0], luminance, 0.73 cd/m^2^) on a gray background (RGB: [128, 128, 128], luminance, 19.51 cd/m^2^). See Appendix S1 for stimuli used in Experiment 1.

**Table 1:** Properties of the Stimuli in Experiments 1 and 2.

Experiment 1									
		Two-word condition				One-word condition			
Character position		1	2	3	4	1	2	3	4
number of strokes	Mean	7.40	7.02	7.54	8.19	7.13	8.31	6.77	7.69
	Sd	2.39	3.07	2.59	2.55	3.10	3.41	2.90	2.98
frequency	Mean	1456	1567	997	892	2214	1369	2668	1492
	Sd	2462	2207	1294	1092	3320	1887	3347	1712
**Experiment 2**									
number of strokes	Mean	7.95	7.97	8.09	7.98	7.85	7.96	8.05	7.92
	Sd	2.56	2.81	2.63	2.84	2.55	2.60	2.60	2.82
frequency	Mean	1070	1057	1027	1057	957	973	1087	1097
	Sd	1280	1109	1264	1086	1328	1298	1498	1244

#### Procedure

A black fixation point was presented at the center of the screen for 500 ms, and subjects were asked to fixate on it. Then the characters were presented for 5 frames (about 83 ms) before they disappeared. Character 1 was presented at the same position as the fixation point. After the Chinese characters disappeared, in 36 of the 48 trials in each condition, a red square (the attention probe, 1°×1° in size, RGB: [255, 0, 0], luminance, 21.72 cd/m^2^) was immediately presented for 2 frames (about 33 ms) equally often at one of the four character locations. Subjects were asked to press one of two keys to indicate whether the red square was present or not.

### Results

#### Accuracy

The overall accuracy (including both target and catch trials) was high (97.6%). In the probe present trials, accuracy did not differ across conditions and across probe positions (*F*s<1), so it was not analyzed further.

#### Reaction time to the probe

Incorrect trials and any trials with a RT shorter than 100 ms or with a RT over three standard deviations from the mean RT (calculated separately for each combination of condition, probe position, and participant) were excluded from analysis. In total, 4.5% of the trials were excluded from analysis.

**Figure 2:**
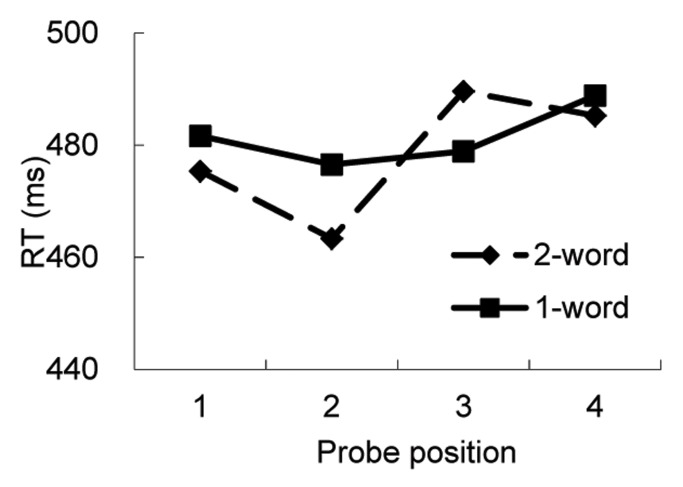
Mean reaction times for probe detection as a function of probe position in Experiment 1.

Mean RT was calculated for each subject at each probe location for each condition. The means across subjects are shown in Figure 2. A 2 (condition) x 4 (probe position) within subject analysis of variance (ANOVA) was conducted. There was a main effect of probe position, *F*(3,117) = 2.70, *p* = .049, MSE = 1696. RT at character 1 position was longer than that at character 2 and 3 positions. Nothing else was significant.

Because we were mainly interested in attention distribution across the word boundary, we conducted a separate analysis with only position 2 and 3. The word boundary in the two-word condition was between these two characters. If word boundaries affect attentional deployment, we expected different RT patterns between the two conditions, and hence an interaction between position and condition. That is, there should be a larger RT difference between positions 2 and 3 in the two-word condition than that in the one-word condition. There was a main effect of position, *F* (1,39) = 4.79, *p* = .035, MSE = 1716, as RT was shorter when the probe was at character 2 position (470 ms, s.e. = 4 ms) than when it was at character 3 position (483 ms, s.e. = 4 ms). More importantly, there was an interaction between condition and position, *F*(1,39) = 5.62, *p* = .023, MSE = 1018. Simple effect analysis showed that in the two-word condition, there was a word boundary effect. RTs were 27 ms shorter when the probe was at the character 2 position than at the character 3 position (467 ms vs. 490 ms), *F* (1,39) = 9.26, *p* = .004, MSE = 1494. However, in the one-word condition, RT was not significantly different when the probe was at character position 2 than when it was at character position 3 (477 ms vs. 479 ms), *F*s<1. Thus, these results confirmed our prediction that word boundaries can constrain visual attention deployment.

## Experiment 2

In Experiment 1, there were only 9 trials for each probe position in each condition, and some of the properties of the characters were not well controlled. These properties, such as character frequency and character complexity, might affect RTs. In Experiment 2, a wider variety of stimuli was used and the properties of the stimuli were more carefully controlled.

### Methods

#### Participants

Forty participants (23 women and 17 men) from the same pool as Experiment 1 participated in this experiment. None of these participants participated in Experiment 1.

#### Apparatus

Stimulus presentation and response registration were controlled by a personal computer. Stimuli were presented on a 21-inch CRT monitor (Sony G520) with a resolution of 1024×768 pixels and a refresh rate of 85 Hz. Participants viewed the stimuli about 70 cm from the monitor.

#### Materials

Thirty practice trials and 256 experimental trials were used in Experiment 2. None of these words was used in Experiment 1. Half of the 256 experimental trials were in the one-word condition, and the other half were in the two-word condition. A probe was presented in 192 of the trials, and no probe was present in the other 64 trials. Some properties of the stimuli are shown in Table 1. The number of strokes and character frequency across conditions were tested with two separate 2 (condition) x 4 (position) ANOVAs. Neither main effect of condition nor position was significant, nor was the interaction between the two factors (*F*s<1). However, we could not control word frequency between the two conditions although we tried our best. According to the published resource (Lexicon of common words in contemporary Chinese, 2009), the frequencies of 4-character words are usually lower than those of 2-character word. The word frequency in the two word condition (0.75 occurrences per million) was higher than that in the one word condition (0.33 occurrences per million), *t*(254) = 10.65, *p*<.001. See Appendix S2 for stimuli used in Experiment 2.

#### Procedure

Procedures were identical to those in Experiment 1 except for the following differences. The characters were presented for 7 frames (about 82 ms), and the probe was presented for 2 frames (about 24 ms). The luminance of characters, background and probe was 0.84 cd/m^2^, 11.79 cd/m^2^,9.29 cd/m^2^ respectively. Participants made responses by pressing a button on a button box, with the left index finger pressing the top button, and the right index finger pressing the bottom button.

### Results

#### Accuracy

Again, the overall accuracy was high (97%). In the probe present trials, accuracy did not differ across conditions and across probe positions (*F*s<1), so it was not analyzed further.

#### Reaction time to the probe

The same method of data trimming was used as in Experiment 1. In total, 3.2% of the target present trials were excluded from analysis.

**Figure 3:**
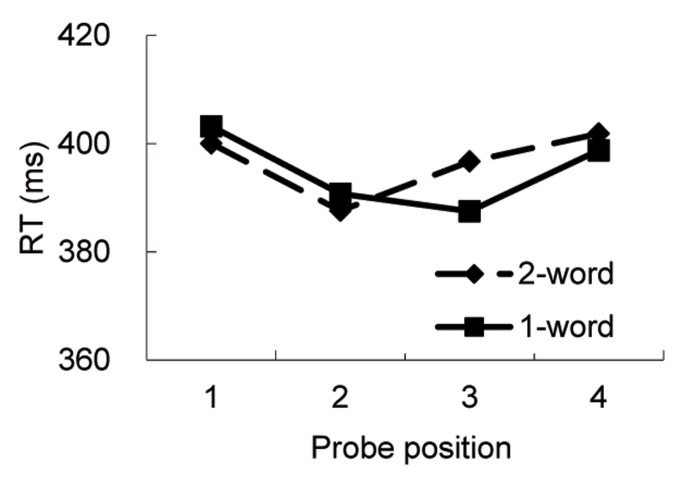
Mean reaction times for probe detection as a function of probe position in Experiment 2.

The means of RT across subjects are shown in Figure 3. As in Experiment 1, Mean RT was calculated for each subject at each probe location for each condition. There was a main effect of probe position, *F* (3,117) = 8.60, *p*<.001, MSE = 342. RT at character 1 position was longer than that at character 2 and 3 positions. Nothing else was significant.

As in Experiment 1, we also conducted a separate analysis for those trials in which the probe was presented at positions 2 and 3. Unlike in Experiment 1, the main effect of position was not significant, *F*(1,39) = 1.27, *p* = .249, MSE = 310. However, most importantly, as in Experiment 1, there was an interaction between condition and position, *F* (1,39) = 6.08, *p* = .026, MSE = 241. Simple effect analysis showed that in the two-word condition, RT was 9 ms shorter when the probe at character position 2 than at character position 3 (387 ms vs. 396 ms), *F*(1,39) = 4.28, *p* = .045, MSE = 323. However, in the one-word condition, RT was 3 ms longer when the probe was at character position 2 than at character position 3 (391 ms vs. 388 ms); but the 3 ms difference was not close to significant, *F* (1,39) = 1.15, *p>.*1. Thus, these results replicated the results of Experiment 1 and suggest that word boundaries can constrain visual attention deployment. Note that RTs were longer in Experiment 1 than in Experiment 2. Three factors might have caused this. First, the CRT monitor used in Experiment 2 had better time properties than the LCD monitor used in Experiment 1. Second, the button box used in Experiment 2 had better time response than the keyboard used in Experiment 1. Third, one finger from each hand was used to press the buttons in Experiment 2, while two fingers in the same hand were used in Experiment 1. This might also slowed down the responses.

## General Discussion

In this study, we examined whether word boundaries affect attentional deployment during Chinese reading. In a probe detection task, we found that RTs were significantly longer when the probe was at character position 3 than at character position 2 in the two-word condition, in which character 2 and character 3 belonged to different words. In contrast, the RTs did not differ that much in the one-word condition, in which characters 2 and 3 belonged to the same word. The results were consistent across the two experiments. The results showed that spatial attention is deployed differently to different sides of word boundary, suggesting that word boundary constrain attentional deployment in Chinese reading.

The word boundary effect of attentional deployment suggests that the word is a major unit of visual attention deployment during reading Chinese, so that the characters belonging to a word are attended together. Because of the holistic properties of word processing, characters that belong to a word are processed as a unit [8]. We theorize that when a reader processes a word, attention is mostly constrained to those characters that constitute the word until it is fully processed. Thus, when the probe was presented at the positions belonging to an attended word, detection responses should be faster than when it was presented at the position that does not belong to the currently attended word.

In the current study, the fixation point was at character position 1. Because of limitations from eccentricity and reading habits, the characters on the left of the display were more likely to be attended to initially and thus attended to when the probe character was presented. Thus, in the two-word condition, most (if not all) of attention was probably focused on the first word on the display when the probe was presented because the exposure of characters was brief. However, in the one-word condition, because there was no reason to limit attention to the first two characters, the RTs did not differ much between probes at character positions 2 and 3. This process is analogous to the findings of object-based attention literature: when part of an object is attended, all of the other parts of the object are also attended to some extent [3], [4].

In Chinese reading, there are no spaces between words. Without spaces, how do Chinese readers segment words and deploy attention within the constraints of word boundaries? It should also be noted that the Chinese word segmentation problem is similar to the speech perception problem in the sense that there are no explicit word boundary markers in the speech stream. The interactive activation model has been successfully used to model speech segmentation [9] via a continuous mapping process. During speech perception, that model assumes that the set of words that best matches the whole string wins the competition. Chinese word segmentation might also work in a similar way. Li et al. [6] proposed a model of Chinese word segmentation based on an interactive activation account [10]. The model assumed that Chinese word recognition is an interactive process involving many nodes at multiple levels (a visual feature level, a letter level, and a word level). Information could feed forward from the lower level to the higher level, and also could feedback from the higher level and affect the processing at the lower level. Characters are processed in parallel (within the constraints of eccentricity) at the character level, but only one single word wins the competition at the word level. When the characters appear in the current experiment, they automatically trigger representations of words that contain those characters. At the same time, activation from the word representation is fed back to influence lower level processing. By this procedure, the characters belonging to a same word are processed as a unit. When a single word wins the competition, the word is recognized, and the word is segmented at the same time. Although the model does not make any assumption on how did the word processing affects attentional deployment, it can be expanded to account for the finding from the present study by assuming that when the activity of the word node reaches a certain level, it can feed back and affect attentional deployment.

**Figure 4:**
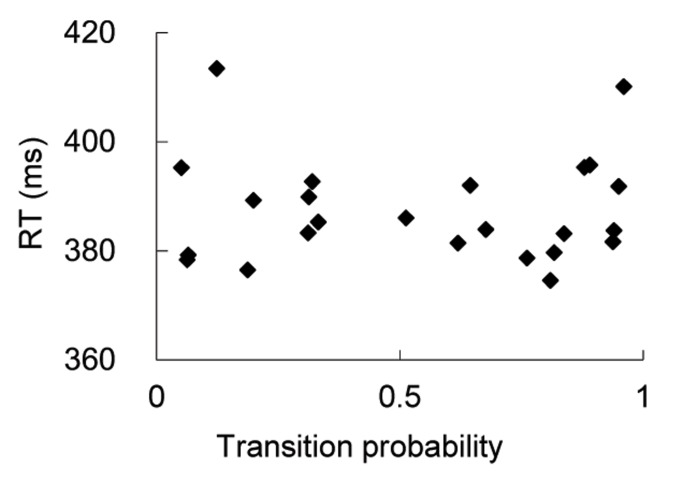
Mean reaction times for probe detection as a function of transition probability of characters in position 3 of one word condition in Experiment 2.

Besides the existence of a word boundary between character 2 and 3, there were some other factors that were different between the two conditions. One factor that differs across the two conditions was the transition probability. For example, in Experiment 2, the transition probability of character 3 given characters 1 and 2 was higher for the one-word condition (.55) than the two-word condition (.01). To test whether transition probability difference alone could cause the RT differences between the two conditions, we examined whether transition probability affects RTs when the probe was at character position 3 in the one-word condition. We did this with the Experiment 2 data since the variance in transition probability was large enough for meaningful analyses. Note that the word boundary effect was reflected mainly with the RT difference at position 3 between the two conditions. If transition probability was a major factor that caused the word boundary effect, we should expect that probe RTs at character 3 should be shorter for larger transition probability items. The scatter plot is shown in Figure 4. The correlation between transition probability and RT was very small (.02) and was not significantly greater than 0 (*t*(39)<1). This suggests that the transition probability is unlikely to be a major factor causing the word boundary effect on attention in our experiments, although it might be one of the factors that Chinese readers use when they segment words.

Another factor that differs between the two conditions is word frequency. As in English, the frequencies of longer Chinese words are usually lower than those of shorter words, making it impossible to balance word frequency between the two conditions. We suspect that word frequency differences cannot explain our data, because most studies on word frequency effects show that high frequency words are usually processed faster than low frequency words [11]. If word frequency affected RT in this study, we would expect that RT should be shorter for the two-word condition since word frequencies were higher in that condition. However, the RT was longer at character position 3 for the two-word condition.

The third factor that differs between the two conditions was the working memory load and task demands. There were two words in the two-word condition, but only one word in the one-word condition. If this factor caused any difference in the RTs, we would expect that the RTs should be longer at all of the probe positions in the two-word condition than in the one-word condition. However, as shown in Figure 3, RTs in the two-word condition were longer that in the one-word condition when the probe was at character position 3, but not when the probes were at the other three positions.

To summarize, we found that RTs increased after a word boundary position in a probe detection task, suggesting that word boundary information affects visual attention deployment during Chinese reading.

## Supporting Information

Appendix S1 Materials in Experiment 1.
(DOCX)

Appendix S2 Materials in Experiment 2.
(DOCX)
