# Supplementary material for: Word Boundaries Affect Visual Attention in Chinese Reading
Source: PLoS One. 2012 Nov 9;7(11):e48905. doi: 10.1371/journal.pone.0048905 (PMC3494710; doi:10.1371/journal.pone.0048905)
Supplement: Appendix S1 — Materials in Experiment 1. (DOCX) [file pone.0048905.s001.docx]

Appendix S1

Materials in Experiment 1

| two-word | one-word |
| --- | --- |
| 有力甜美 | 始料未及 |
| 崎岖坦诚 | 名副其实 |
| 齐全格外 | 美中不足 |
| 阴暗非法 | 耳熟能详 |
| 冰冷大声 | 全神贯注 |
| 真心纤细 | 突飞猛进 |
| 妥当奇异 | 大智若愚 |
| 急速切实 | 焕然一新 |
| 恰当密闭 | 与众不同 |
| 过多活络 | 不约而同 |
| 惊人未免 | 脍炙人口 |
| 偏远巧妙 | 锲而不舍 |
| 无关鲜美 | 突如其来 |
| 总共纯真 | 信以为真 |
| 邪恶由衷 | 无庸置疑 |
| 连串差点 | 顺理成章 |
| 迥异阳刚 | 运筹惟幄 |
| 虚心私密 | 五花八门 |
| 辛勤单调 | 一触即发 |
| 冗长再次 | 家喻户晓 |
| 动感低迷 | 千变万化 |
| 优先日益 | 顾名思义 |
| 客气全心 | 粉墨登场 |
| 富有仍旧 | 不由自主 |
| 亲手无情 | 相辅相成 |
| 扎实轻盈 | 首当其冲 |
| 神勇每次 | 锦上添花 |
| 有利详尽 | 不可或缺 |
| 平易以来 | 乐此不疲 |
| 稀有如意 | 举足轻重 |
| 辛劳心跳 | 不遗余力 |
| 心酸艰辛 | 不知所措 |
| 贫乏起码 | 与生俱来 |
| 自豪强壮 | 并驾齐驱 |
| 门外突发 | 追根究底 |
| 诚恳顺畅 | 推陈出新 |
