# Supplementary material for: Word Boundaries Affect Visual Attention in Chinese Reading
Source: PLoS One. 2012 Nov 9;7(11):e48905. doi: 10.1371/journal.pone.0048905 (PMC3494710; doi:10.1371/journal.pone.0048905)
Supplement: Appendix S2 — Materials in Experiment 2. (DOCX) [file pone.0048905.s002.docx]

Appendix S2

Materials in Experiment 2

| two-word | one-word |
| --- | --- |
| 气量合群 | 得过且过 |
| 清仓憋气 | 形单影只 |
| 叙说脉象 | 相敬如宾 |
| 仁德叹服 | 若即若离 |
| 斧头骑术 | 深居简出 |
| 退步还俗 | 奉公守法 |
| 粗心惨白 | 虎落平阳 |
| 悔过反衬 | 喜新厌旧 |
| 贺信劲风 | 有惊无险 |
| 思虑表针 | 煽风点火 |
| 经脉立论 | 毁誉参半 |
| 插队光鲜 | 好高骛远 |
| 官服感化 | 殊途同归 |
| 安检昏话 | 文过饰非 |
| 辈分高估 | 高朋满座 |
| 保质机敏 | 有求必应 |
| 民防宿命 | 情同手足 |
| 交心池水 | 因材施教 |
| 保送清净 | 将功赎罪 |
| 清纯记性 | 吐故纳新 |
| 安祥赤身 | 张皇失措 |
| 日食空乏 | 目无法纪 |
| 代销纤美 | 洗耳恭听 |
| 明净方桌 | 鬼使神差 |
| 剑术海拨 | 香消玉殒 |
| 缆车禅理 | 得陇望蜀 |
| 发呆禅机 | 言传身教 |
| 提神呈报 | 意味深长 |
| 调档扯开 | 奇货可居 |
| 参透皎月 | 先斩后奏 |
| 珍重拉练 | 以柔克刚 |
| 多虑传唱 | 心领神会 |
| 布署妙计 | 见微知著 |
| 偶而方框 | 昂首阔步 |
| 分派捧场 | 养尊处优 |
| 道观白斑 | 老当益壮 |
| 当差拳术 | 安贫乐道 |
| 秀气发迹 | 负荆请罪 |
| 法号年货 | 受宠若惊 |
| 对答埋没 | 药到病除 |
| 扮相打盹 | 名正言顺 |
| 反语放哨 | 平心而论 |
| 谦和递进 | 指桑骂槐 |
| 逆反谈资 | 阳奉阴违 |
| 娇美便车 | 任重道远 |
| 弃权和顺 | 怵目惊心 |
| 钓具白痴 | 好逸恶劳 |
| 文痞真迹 | 见机行事 |
| 采办沸水 | 夜以继日 |
| 拆线保底 | 挥洒自如 |
| 企求盲点 | 虚怀若谷 |
| 神化条码 | 刨根问底 |
| 出迎搬走 | 变废为宝 |
| 合脚赔本 | 如花似玉 |
| 生疏命数 | 举世闻名 |
| 民谣方块 | 无足轻重 |
| 求购对调 | 点到为止 |
| 代劳比照 | 身经百战 |
| 生肖禅学 | 沽名钓誉 |
| 禅思常理 | 从善如流 |
| 类同分封 | 知书达礼 |
| 饯行挪动 | 安居乐业 |
| 盲文军训 | 徒有虚名 |
| 并拢问路 | 闻鸡起舞 |
| 逆行理赔 | 画蛇添足 |
| 传承戒心 | 固若金汤 |
| 叠加争抢 | 物以类聚 |
| 秘方面议 | 开诚布公 |
| 窘相拨开 | 量体裁衣 |
| 地皮金榜 | 交头接耳 |
| 刮风就范 | 秋高气爽 |
| 电扇文豪 | 见贤思齐 |
| 求签对味 | 随遇而安 |
| 畅行斥资 | 暗送秋波 |
| 把柄插头 | 古往今来 |
| 盗用恒心 | 随声附和 |
| 出诊道袍 | 登峰造极 |
| 夯实法老 | 隔墙有耳 |
| 果木清秀 | 眉清目秀 |
| 调侃地契 | 物极必反 |
| 动粗统管 | 瓜熟蒂落 |
| 赌资舱位 | 饱食终日 |
| 地洞碰面 | 冒名顶替 |
| 合谋家室 | 匪夷所思 |
| 文静搅合 | 各奔前程 |
| 参股娇好 | 唇枪舌剑 |
| 面额狠心 | 捕风捉影 |
| 从众出借 | 流芳百世 |
| 涂改定稿 | 鹿死谁手 |
| 铺开拘传 | 恶贯满盈 |
| 权位拆台 | 韬光养晦 |
| 盲从领班 | 引火烧身 |
| 扇动白净 | 风烛残年 |
| 畅想砍价 | 强词夺理 |
| 弹出苍生 | 浅尝辄止 |
| 剪发反串 | 空前绝后 |
